# Association of Serum Calcium With Infarct Size and Severity in Acute Ischemic Stroke: A Rural Hospital-Based Cross-Sectional Study

**DOI:** 10.7759/cureus.43015

**Published:** 2023-08-06

**Authors:** Nipun Bawiskar, Sunil Kumar, Sourya Acharya, Nirmesh Kothari, Rinkle R Gemnani

**Affiliations:** 1 Medicine, Jawaharlal Nehru Medical College, Datta Meghe Institute of Medical Science (Deemed to be University), Wardha, IND

**Keywords:** albumin corrected calcium, ionic calcium, total calcium, barthel index, nihss, infarct size, stroke

## Abstract

Background

One of the major mediators of ischemic neuronal cell death is calcium. It has been found that elevated serum calcium is associated with a better prognosis in patients with ischemic stroke. This study highlights the association of serum calcium, albumin-corrected calcium, and ionic calcium with the size of acute ischemic stroke as well as severity outcome in terms of the National Institutes of Health Stroke Scale (NIHSS) score and Barthel Index.

Methods

This cross-sectional study was conducted on 85 cases of acute ischemic stroke (based on a computerized tomography scan of the brain) from September 2019 to October 2021. All included patients had undergone complete clinical history, systemic examination, as well as estimation of serum total calcium, albumin corrected calcium, and ionic calcium. NIHSS score and Barthel Index were used to access the severity of each subject.

Results

A significant positive correlation was seen between infarct size with NIHSS with a correlation coefficient of 0.35. A significant negative correlation was seen between infarct size with serum calcium, albumin-corrected calcium, and Barthel Index with a correlation coefficient of -0.483, -0.354, and -0.365 respectively. No correlation was seen between infarct size and ionic calcium with a correlation coefficient of 0.082.

Conclusion

It can be concluded that higher normal levels of serum calcium and albumin-corrected calcium are associated with a smaller-sized infarct and had less severity index among patients with acute ischemic stroke.

## Introduction

A stroke is a cerebrovascular event with signs and symptoms consistent with cerebral dysfunction. These develop rapidly over twenty-four hours and may result in chronic debility or mortality [1]. Occlusion in the cerebral blood flow due to a thrombus or embolus leads to ischemic stroke. Atheroma, cardioembolic phenomenon, and artery-to-artery embolism are some of the causes that lead to decreased blood flow [2]. Stroke presents in two regions: the inner ischemic core and the surrounding area of hypoperfusion, the penumbra [3]. Certain risk factors may be associated with stroke like diabetes mellitus, hypertension, hyperhomocysteinemia, raised levels of c-reactive protein, and uric acid [4].

The normal cerebral blood flow in humans is 45-50 ml/min/100 gm. When this rate decreases to 10ml/min/100gm depolarization of neurons occurs along with disturbances like rapid loss of intracellular potassium, reduction of adenosine triphosphate, and entry of calcium and sodium into the cells. Calcium influx occurs secondary to interruption in the O2-dependent generation of high-energy compounds. Calcium overload into the mitochondria causes a decrease in oxidative phosphorylation. There is the activation of membrane phospholipases and protein kinase which in turn causes the formation of free fatty acid [5]. Activation of the inflammatory cascade with mediators like prostaglandin, leukotrienes, and free radicals causes denaturation of intracellular proteins and enzymes. Thus, calcium homeostasis plays an important role in acute ischemic stroke [6,7].

Serum calcium is found as ionic calcium, protein-bound calcium, and in complex form. It binds to both albumin and globulin. This is about 40 % of the total calcium in the body. Calcium when present in complex form is absorbed by various tissues and is distributed throughout the body as calcium carbonate, calcium phosphate, and calcium oxalate [8]. Ionic calcium is free calcium that is utilized by the body for different functions and this account for 51% of total body calcium. Higher albumin-corrected calcium levels were directly associated with severity in terms of neurological outcomes like morbidity and mortality after acute ischemic stroke. Serum calcium also correlates with the size of cerebral infarction and clinical outcomes [9,10]. This study highlights the association of serum calcium, albumin-corrected calcium, and ionic calcium with the size of acute ischemic stroke as well as severity outcome in terms of the National Institutes of health stroke scale (NIHSS) Score and Barthel Index.

## Materials and methods

This cross-sectional study was performed on 85 patients with acute ischemic stroke from September 2019 to October 2021 after institutional ethical committee approval.

**Figure 1:**
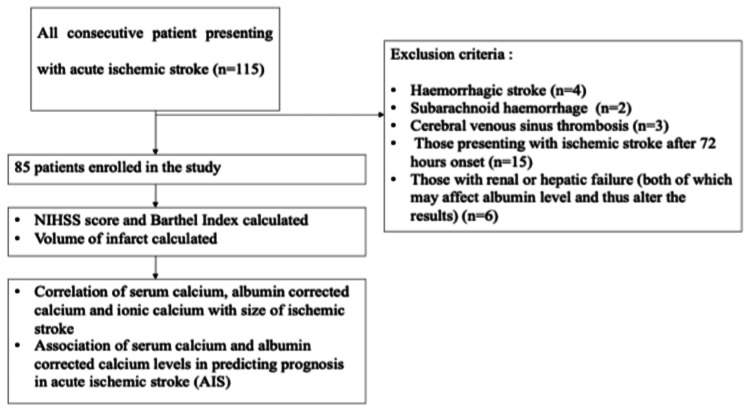
Flow chart of the study.

Detailed clinical history and examination were done and investigations specific to the study like complete hemogram, renal function test, liver function test, lipid profile, serum calcium, ionic calcium, and computed tomography (CT) of the brain were performed. Patients with acute ischemic stroke aged >18 years who were diagnosed within the previous 72 hours by examination and confirmed by a CT scan were included in the study. Patients with hemorrhagic stroke, subarachnoid hemorrhage, cerebral venous sinus thrombosis, those presenting with ischemic stroke after 72 hours of onset, and those with renal or hepatic failure (both of which may affect albumin level and thus alter the results) were excluded from the study. The flow chart of the study is shown in Figure 1.

Hemogram was obtained from a peripheral venous sample collected in an ethylenediamine tetraacetic acid (EDTA) bulb on admission and was run using an automated machine- UnicelDxH 800 Coulter Cellular Analysis System (Beckman Coulter), Danaher Corporation Company, Brea, California, United States.

Ionic calcium was estimated using an automated blood gas analyzer. Albumin-corrected calcium level was calculated as the sum of serum total calcium level and 0.8 times the difference between the normal population albumin level (4 mg/dl) and the patient’s albumin level. Albumin-corrected calcium level = serum total calcium level + 0.8 × (4 − patient's albumin level).

CT imaging was performed to calculate the size of the infarct, and the largest slice in the affected area was selected. A ruler tool was used to measure the longest axis in the lesion which was labeled as the x [A] axis. Another line was drawn perpendicular to it at the widest dimension and was labeled as the y [B] axis. The third axis was calculated by multiplying the number of slices with slice thickness and was the z [C] axis. The x, y, and z axis were calculated in mm. The infarct size was then calculated by ABC/2 which has been deemed the most accurate for calculating the size of the ischemic stroke [8]. The infarct size as used in the study was in cubic mm.

Outcome measures

The primary outcome of the study was to study serum calcium, ionic calcium, and albumin-corrected calcium in relation to the size of the ischemic stroke. The secondary outcome was to study the severity in terms of NIHSS score and Barthel Index about the size of ischemic stroke.

Statistical analysis

Statistical analysis was done using the Spearman rank correlation coefficient. The final analysis was done with the use of Statistical Package for Social Sciences (SPSS) software (IBM Corp. Released 2012. IBM SPSS Statistics for Windows, Version 21.0. Armonk, NY: IBM Corp). For statistical significance, a p-value of less than 0.05 was considered statistically significant.

## Results

Among 85 patients, the mean age of study subjects was 58.6 ± 13.8 years. 8.24% patients had brainstem infarct, 3.53% had cerebellar infarct, 11.76% had an infarct in the corona radiata, 12.94% had an infarct in ganglio-capsular region, 5.88% had an infarct in the internal capsule, 3.53% had infarct in thalamus and 54.12% had cortical infarcts. The mean value of serum calcium (mg/dl) was 8.96 ± 0.91, albumin-corrected calcium was 9.17 ± 0.92 and ionic calcium was 4.35 ± 0.53. Other baseline characteristics are shown in Table 1.

**Table 1:** Baseline characteristics and baseline profile of the study subjects.

Parameter	Percentage/ Mean
Age	
Mean Age ± SD	58.6 ± 13.8
18-30	3.53%
31-60	48.24%
>60	48.24%
Gender	
Female	35.29%
Male	64.71%
Common clinical presentations	
Right-sided Hemiparesis	36.5%
Left-sided Hemiparesis	33%
Abnormal Body movements	2.4%
Drowsy/ loss of consciousness	5.8%
Giddiness and ataxia	1.2%
Sudden loss of vision/ Diminution of vision	2.4%
Altered sensorium	5.8%
Headache	3.5%
Speech disorders and deviation of angle of mouth	8.2%
No weakness	1.2%
Total	100.00%
Mean NIHSS and Barthel Index on admission	
NIHSS	12.07 ± 4.7
Barthel Index	74.41 ± 17.24
Co-morbidities	
Diabetes mellitus	20.00%
Hypertension	56.47%
Others Ischemic heart disease Rheumatic heart disease Chronic obstructive pulmonary disease Old case of pulmonary tuberculosis)	9.42%
More than 1 co-morbidity	18.82%
Area involved	
Lobar	54.12%
Combined lesion	34.12%
Frontal region	7.06%
Occipital	2.35%
Parietal	10.59%
Brainstem	8.24%
Cerebellum	3.53%
Corona radiata	11.76%
Gangliocapsular region	12.94%
Internal capsule	5.88%
Thalamus	3.53%
Total	100.00%
Lipid profile	
Total cholesterol(mg/dL)	170.71 ± 44.09
LDL (mg/dL)	111.66 ± 39.01
HDL (mg/dL)	38.04 ± 13.02
Triglycerides(mg/dL)	126.48 ± 57.74
Risk Factors:	
Smoking	40.00%
Alcohol	34.12%
Serum calcium, albumin-corrected calcium, ionic calcium	
Serum calcium(mg/dL)	8.96 ± 0.91
Albumin corrected calcium(mg/dL)	9.17 ± 0.92
Ionic calcium(mg/dL)	4.35 ± 0.53

**Figure 2:**
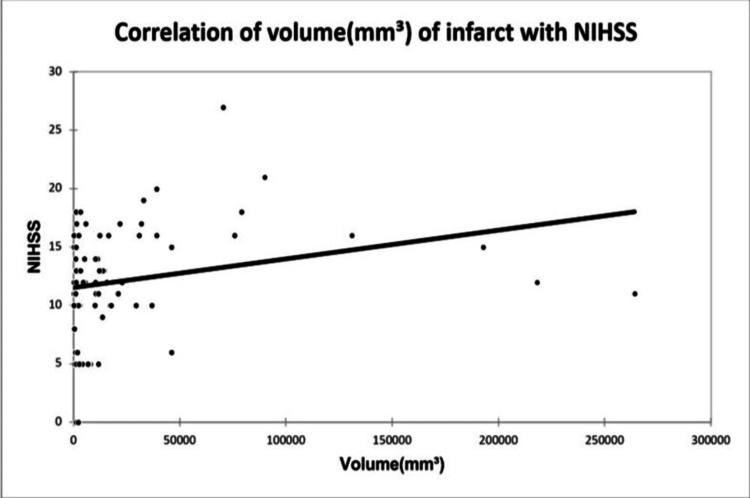
Correlation of the volume of infarct with NIHSS score.

**Figure 3:**
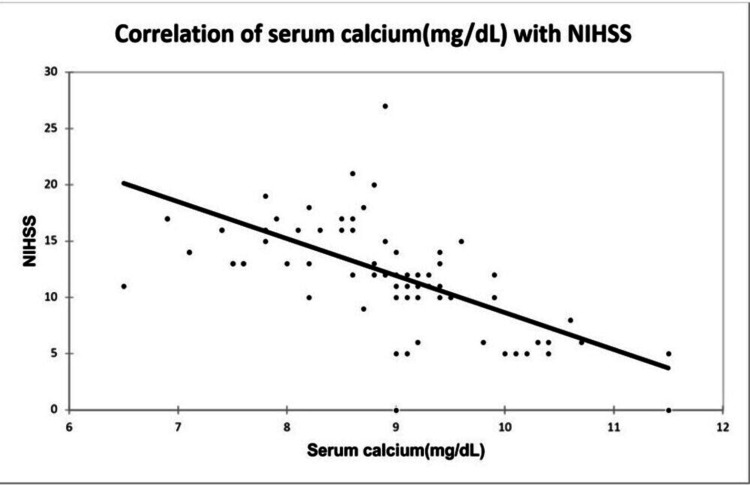
Correlation of serum calcium with NIHSS score.

**Figure 4:**
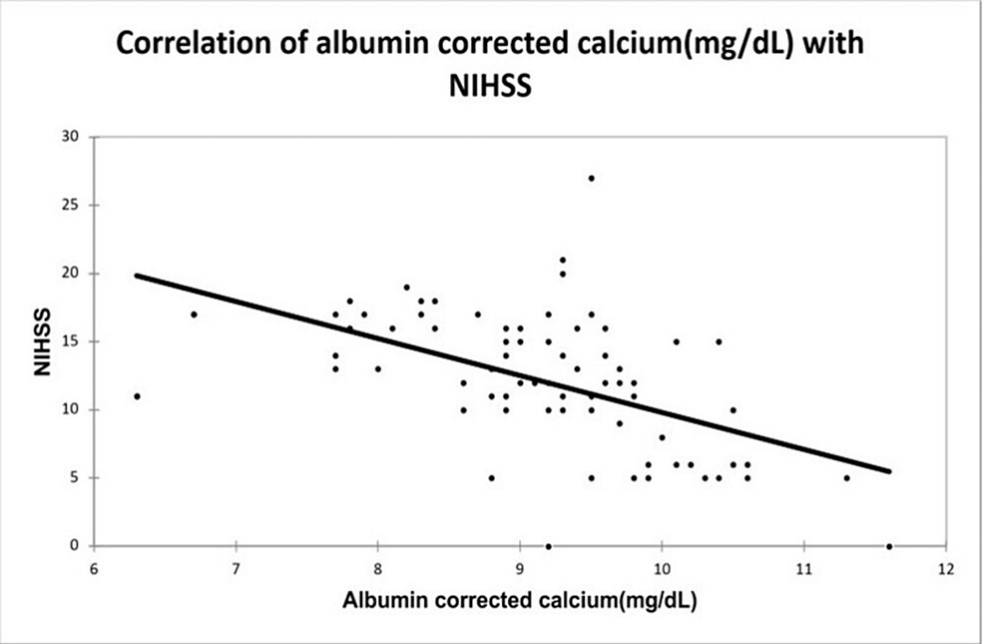
Correlation of albumin-corrected calcium with NIHSS score.

**Figure 5:**
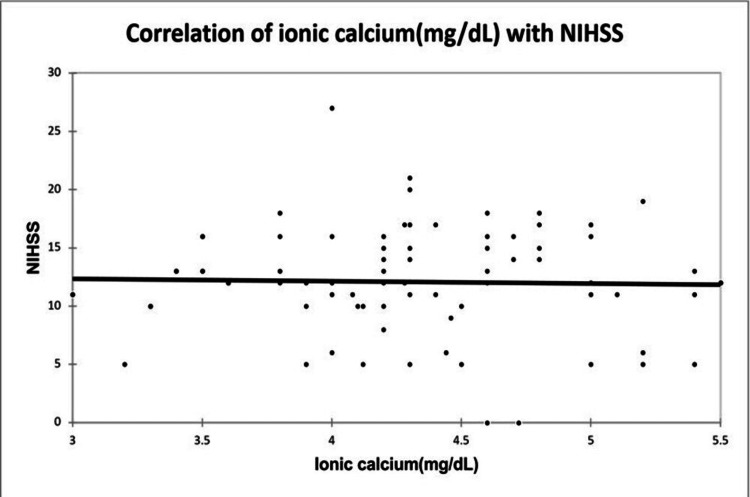
Correlation of ionic calcium with NIHSS score.

**Figure 6:**
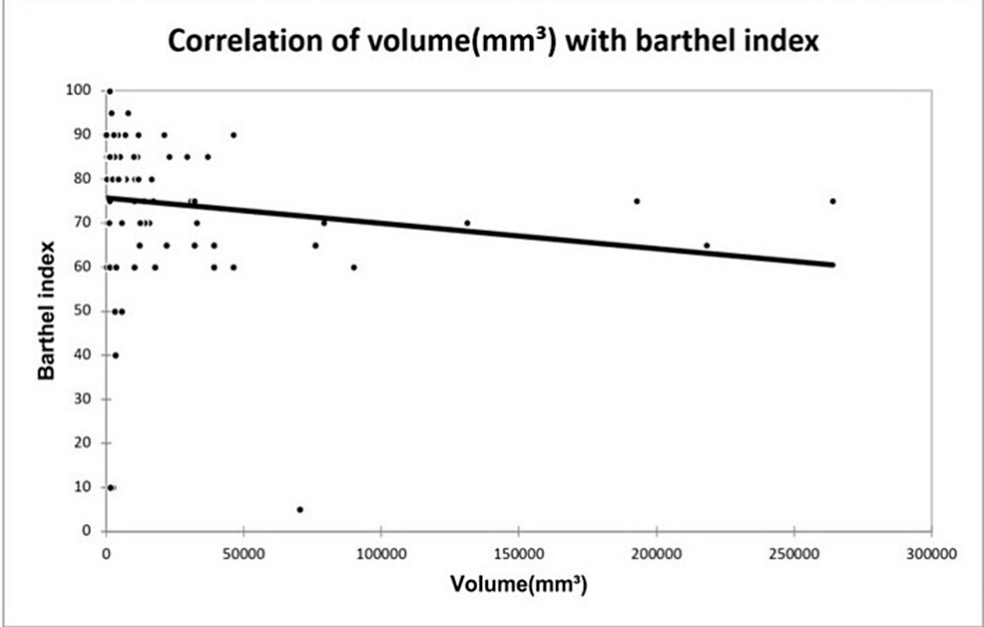
Correlation of volume of infarct with Barthel Index.

**Figure 7:**
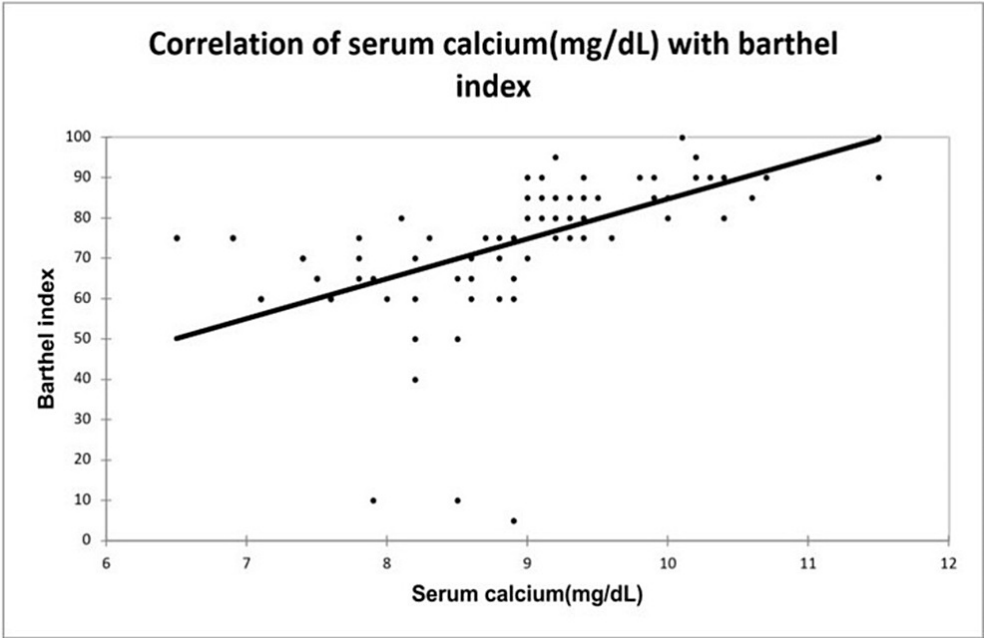
Correlation of serum calcium with Barthel Index.

**Figure 8:**
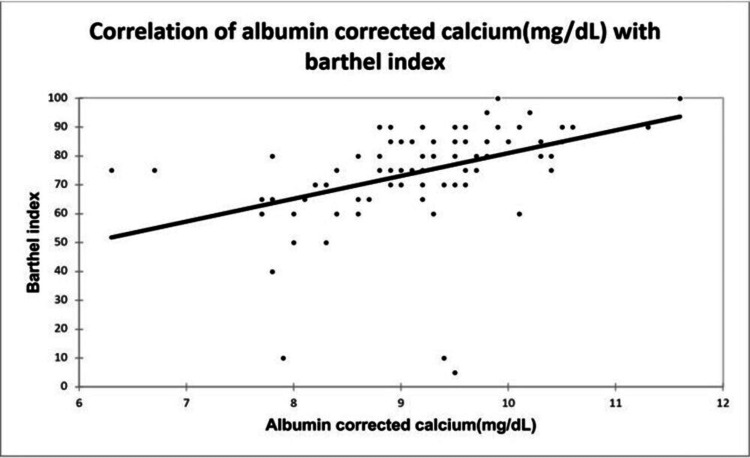
Correlation of albumin-corrected calcium with Barthel Index.

**Figure 9:**
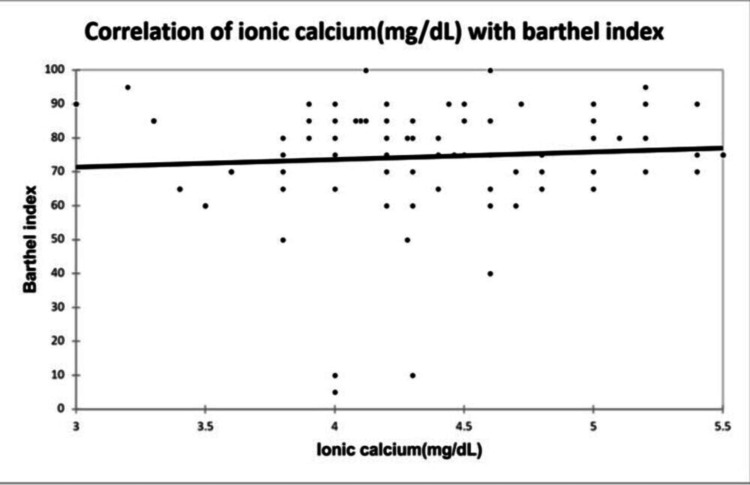
Correlation of ionic calcium with Barthel Index.

A significant positive correlation was seen between NIHSS with infarct size (mm³) with a correlation coefficient of 0.35. A significant negative correlation was seen between NIHSS with serum calcium and albumin-corrected calcium with a correlation coefficient of -0.713, and -0.556 respectively. No correlation was seen between NIHSS with ionic calcium with a correlation coefficient of 0.053. A significant positive correlation was seen between the Barthel Index with serum calcium, albumin corrected calcium with a correlation coefficient of 0.779, and 0.566 respectively. A significant negative correlation was seen between the Barthel Index with infarct size with a correlation coefficient of -0.365. No correlation was seen between the Barthel Index with ionic calcium with a correlation coefficient of 0.002 as shown in Figures 2, 3, 4, 5 demonstrating a correlation with the NIHSS score, and Figures 6, 7, 8, 9 demonstrating a correlation with Barthel Index.

**Figure 10:**
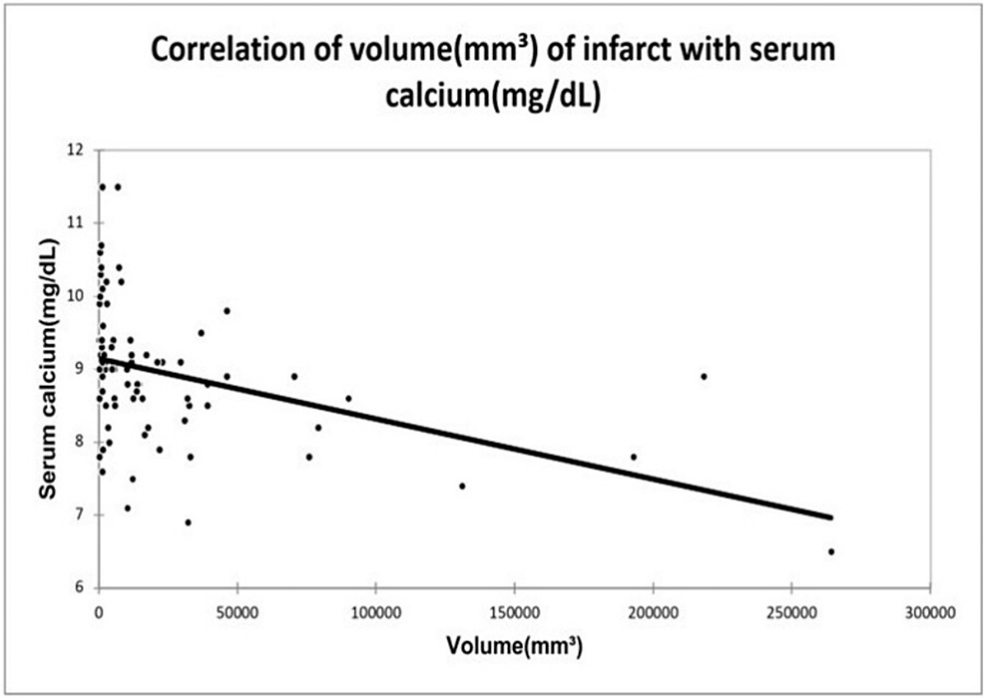
Correlation of volume of infarct with serum calcium.

**Figure 11:**
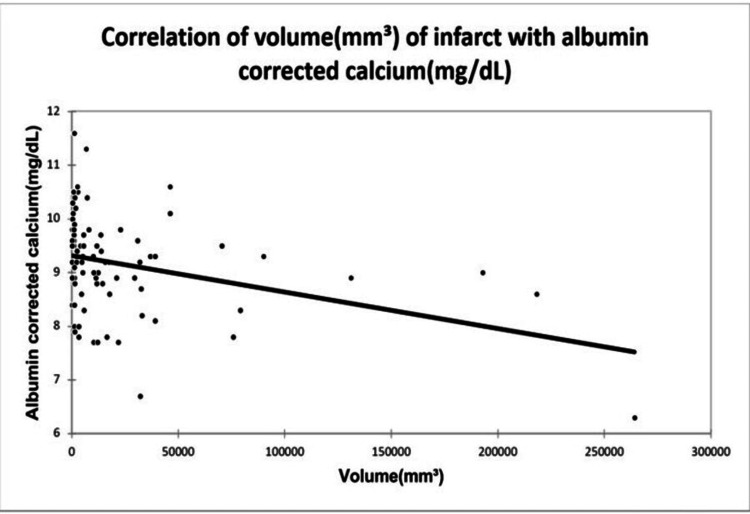
Correlation of volume of infarct with albumin-corrected calcium.

**Figure 12:**
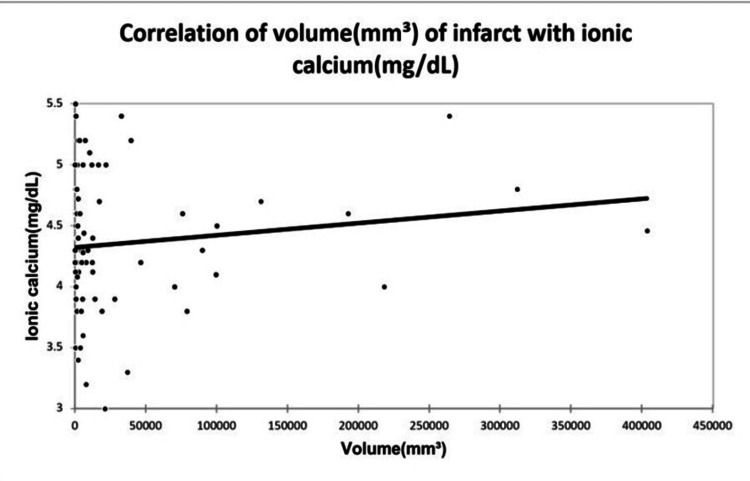
Correlation of volume of infarct with ionic calcium.

A significant positive correlation was seen between infarct size with NIHSS with a correlation coefficient of 0.35. A significant negative correlation was seen between infarct size with serum calcium, albumin-corrected calcium, and Barthel Index with a correlation coefficient of -0.483, -0.354, and -0.365 respectively. No correlation was seen between infarct size and ionic calcium with a correlation coefficient of 0.082 as shown in Figures 10, 11, 12.

## Discussion

In our study, we found that higher normal levels of serum calcium and albumin-corrected calcium were associated with a smaller-sized infarct and better prognosis in terms of NIHSS and Barthel Index among patients with acute ischemic stroke. Ionic calcium did not correlate with the size of the infarct or the prognosis of the patients.

Ischemic stroke occurs following critical stenosis in the cerebral circulation leading to a decrease in blood flow and ischemia. Hypoxia leads to disturbance in cellular homeostasis and movement of calcium from extracellular space into the cells. This leads to enzyme cascade activation and lipid peroxidation further increasing the influx of calcium into the cells. Ovbiagele et al. conducted a study in 2008 on 826 patients on patients with acute ischemic stroke and found that increased levels of serum Ca predict higher independence three months following ischemic stroke [10]. They also found that very early serum calcium appears not to have any prognostic significance, whereas in our study higher normal levels of serum calcium and albumin-corrected calcium were associated with a smaller-sized infarct and better prognosis in terms of NIHSS and Barthel Index. Buck et al. in 2007 found that the infarct size was comparatively smaller in patients with higher levels of serum calcium on admission [6]. Appel et al. performed a study in 2011 on 784 and found that serum calcium was a marker of mortality among patients with ischemic stroke [11]. Albumin-corrected calcium was the only factor to be associated with long-term mortality. Suryawan et al. in 2017 found that patients with poor outcomes had lower levels of serum-adjusted calcium [12,13,14]. A retrospective study done by Chung et al. in 2014 found that there was a significant association between serum calcium and albumin-corrected calcium as linear variables [15,16]. There was no significant association of calcium with NIHSS and Barthel Index as noted by Sivasubramaniyam et al. in 2017. Ishfaq et al. in their study of 138 patients revealed that low levels of serum calcium may be related to more severe clinical findings at the stroke onset [14,17]. A study by Borah et al. revealed that total calcium, albumin-corrected calcium, and ionized calcium had a statistically significant negative correlation with infarct size [5,18].

Strength of the study 

Our study correlated biochemical serum calcium, albumin-corrected calcium, and ionic calcium along with radiological parameters. Functional and clinical indicators such as The National Institute of Health Stroke Scale and Barthel Index were included in this study.

Limitations

Potential confounding factors like socioeconomic status, dietary pattern, etc. were not adjusted. The study was done on a small sample size and the statistical association needs a larger sample size for further validation.

## Conclusions

It can be concluded that higher levels of serum calcium and albumin-corrected calcium are associated with a smaller-sized infarct and better prognosis among patients with acute ischemic stroke. Serum calcium, albumin-corrected calcium, and ionic calcium are often overlooked or not assessed in patients with acute ischemic stroke. Thus, efforts must be made to create awareness with regard to the use of serum calcium in assessing the size of ischemic stroke and its prognostic significance. A multi-centric study with a larger number of patients should be done in order to further assess the role of serum calcium, albumin-corrected calcium, and ionic calcium in acute ischemic stroke.
